# Photobiomodulation for the treatment of neuroinflammation: A systematic review of controlled laboratory animal studies

**DOI:** 10.3389/fnins.2022.1006031

**Published:** 2022-09-20

**Authors:** Fabrízio dos Santos Cardoso, Farzad Salehpour, Norberto Cysne Coimbra, Francisco Gonzalez-Lima, Sérgio Gomes da Silva

**Affiliations:** ^1^Departamento de Farmacologia, Faculdade de Medicina de Ribeirão da Universidade de São Paulo (FMRP-USP), Ribeirão Preto, SP, Brazil; ^2^Department of Psychology and Institute for Neuroscience, University of Texas at Austin, Austin, TX, United States; ^3^Centro Universitário UNIFAMINAS (UNIFAMINAS), Muriaé, MG, Brazil; ^4^Hospital do Câncer de Muriaé, Fundação Cristiano Varella (FCV), Muriaé, MG, Brazil

**Keywords:** photobiomodulation, low-level laser (light) therapy, brain, neuroinflammation, inflammation, cytokine, microglia

## Abstract

**Background:**

Neuroinflammation is a response that involves different cell lineages of the central nervous system, such as neurons and glial cells. Among the non-pharmacological interventions for neuroinflammation, photobiomodulation (PBM) is gaining prominence because of its beneficial effects found in experimental brain research. We systematically reviewed the effects of PBM on laboratory animal models, specially to investigate potential benefits of PBM as an efficient anti-inflammatory therapy.

**Methods:**

We conducted a systematic search on the bibliographic databases (PubMed and ScienceDirect) with the keywords based on MeSH terms: photobiomodulation, low-level laser therapy, brain, neuroinflammation, inflammation, cytokine, and microglia. Data search was limited from 2009 to June 2022. We followed the Preferred Reporting Items for Systematic Reviews and Meta-Analyses (PRISMA) guideline. The initial systematic search identified 140 articles. Among them, 54 articles were removed for duplication and 59 articles by screening. Therefore, 27 studies met the inclusion criteria.

**Results:**

The studies showed that PBM has anti-inflammatory properties in several conditions, such as traumatic brain injury, edema formation and hyperalgesia, ischemia, neurodegenerative conditions, aging, epilepsy, depression, and spinal cord injury.

**Conclusion:**

Taken together, these results indicate that transcranial PBM therapy is a promising strategy to treat brain pathological conditions induced by neuroinflammation.

## Introduction

Neuroinflammation is a response that involves cells of the central nervous system (CNS) such as neurons, macroglia and microglia (DiSabato et al., 2016; Schain and Kreisl, 2017; Shabab et al., 2017). This response is mainly mediated by cytokines, chemokines, secondary messengers, and reactive oxygen species (ROS) (Glass et al., 2010; Park et al., 2011; DiSabato et al., 2016; Norden et al., 2016). Neuroinflammation also can be a pathological condition in a variety of neurodegenerative diseases ((Schain and Kreisl, 2017)). For example, the activation of microglia, pro-inflammatory cytokines and signaling pathways linked to inflammation such as nuclear factor kappa-light-chain-enhancer of activated B cells (NF-κB) pathway can trigger neurodegeneration (Glass et al., 2010; Harry and Kraft, 2012; Lyman et al., 2014). The prolonged release of pro-inflammatory mediators such as tumor necrosis factor-alpha (TNF-α), interleukin (IL)-1α, IL-1β, and IL-6 allow leukocytes to migrate into the brain and induce pathogenesis in the CNS (De Vries et al., 1996; Laflamme et al., 1999). In addition, this inflammatory reaction leads to synaptic gene dysregulation, tissue damage, and potentially cell death (Cunningham et al., 1996; Carson et al., 2006; DiSabato et al., 2016). Given this scenario, new therapeutic approaches are needed to modulate neuroinflammatory responses in pathological conditions.

Photobiomodulation (PBM), or low-level laser/light therapy (LLLT) (Anders et al., 2015), is a non-invasive light-driven intervention that involves the use of red and near-infrared (NIR) light to stimulate healing processes, reduce pain, protect the aging brain and decrease inflammation in several tissues, including the nervous tissue (Rojas and Gonzalez-Lima, 2011, 2013; Almeida et al., 2013; Arany, 2016; Hamblin, 2017; Cardoso et al., 2021a,b). These effects may be mediated by multiple mechanisms. However, cytochrome c oxidase (CCO), the fourth enzyme complex in the electron transport chain within mitochondria, is the main photoacceptor when cells are irradiated with the red to NIR light used for PBM (Karu, 1999). Cellular studies have also shown that PBM promotes ATP synthesis in mitochondria (Karu et al., 1995), and release of mitochondrial ROS and nitric oxide (Karu et al., 2005; Huang et al., 2009). These upstream processes contribute to increased cellular metabolism, altered mitochondrial dynamics, increased vasodilation, and mainly decreased inflammation (Pastore et al., 1994; Karu et al., 2005; Muili et al., 2012; Plass et al., 2012). In the brain *in vivo*, a primary PBM mechanism has been confirmed to be photonic oxidation of mitochondrial CCO (Wang et al., 2017a; Saucedo et al., 2021), being this mechanism independent of heat/thermal effects induced by light (Wang et al., 2017b). Brain PBM leads secondarily to increased cerebrovascular oxygenation (Tian et al., 2016; Holmes et al., 2019), the activation of metabolic pathways (Cardoso et al., 2021c), and of intracellular signaling molecules (Cardoso et al., 2021d), some of them relevant for inflammation (Cardoso et al., 2021a). The mitochondrial mechanism of PBM may provide a link between PBM and inflammation considering that recent studies have uncovered mitochondrial molecules, called mitochondrial alarmins, with inflammatory signaling properties (Grazioli and Pugin, 2018).

In favor of idea, it has been noted that PBM can alter the levels of inflammatory mediators in various animal models (Gupta et al., 2015; Martins et al., 2016; Yoshimura et al., 2016). For example, Gupta et al. (2015) demonstrated that 904 nm laser PBM enhances the healing of burn wounds in rats and attenuates inflammation by decreasing the expression of TNF-α and NF-kB, and by up-regulated expression of VEGF, FGFR-1, HSP-60, and HIF-1α at 4- and 7-days post-wounding. Martins et al. (2016) administered 950 nm laser PBM therapy in an animal model of inflammatory pain and found that the animals exhibited a reduced pain and an improvement of antioxidant enzymes and high levels of the anti-inflammatory cytokine IL-10. In a mouse model of obesity and type 2 diabetes mellitus, six sessions of 830 nm laser PBM were also able to reduce abdominal adipose tissue inflammation (Yoshimura et al., 2016).

In recent years, promising evidence has emerged to support the anti-inflammatory effects of the PBM therapy in various animal models in different neurological conditions (Khuman et al., 2012; Hamblin, 2017; Salehpour et al., 2019a,b; Cho et al., 2020; Cardoso et al., 2021a; Yang et al., 2021a). In this systematic review, we analyzed the neuroinflammatory effects of PBM on animal models of brain pathological conditions, in special to investigate potential translational benefits of PBM as an anti-inflammatory transcranial therapy.

## Materials and methods

### Data sources and search strategy

The search was conducted from 2009 to June 2022. PubMed and ScienceDirect were searched electronically with the keywords “photobiomodulation” or “low-level laser therapy” or “LLLT”; and “brain”; and “inflammation” or “cytokine” or “microglia” or “neuroinflammation” (Table 1). To ensure the clarity and transparency of the articles, we used the Preferred Reporting Items for Systematic Reviews and Meta-Analyses (PRISMA) guidelines (Moher et al., 2010). Two independent researchers screened the title, abstract, and the full text of the articles and judged the searched materials against the inclusion and exclusion criteria. Disagreements were resolved by consensus.

**Table 1:** Summary of laboratory animal studies on neuroinflammatory effects of the photobiomodulation.

**Boolean builder**	**Mesh terms**
	“Photobiomodulation” or “Low-Level Laser Therapy” or “LLLT”
And	“Brain”
And	“Inflammation” or “Cytokines” or “Microglia” or “Neuroinflammation”

### Selection criteria

We selected all *in vivo* studies to obtain findings related to neuroinflammatory effect of PBM in brain disorders (Hamblin, 2017). The search strategy included experimental *in vivo* animal studies conducting PBM. We accepted only publications written in English. Neither *in vitro* approaches, clinical original articles, conference papers, nor review articles were included.

### Data extraction and data synthesis

The included articles were divided according to pathological conditions. For data extraction, groups were subdivided according to the references (author and year), characteristics of the population (animals/species, sex and age), PBM parameters: light source/wavelength, continuous wave (CW) or pulsing mode, output power, irradiance per session, irradiation time, fluence per session, energy per session, irradiation approach/site, number of treatment sessions, and studies outcomes.

## Results

**Figure 1:**
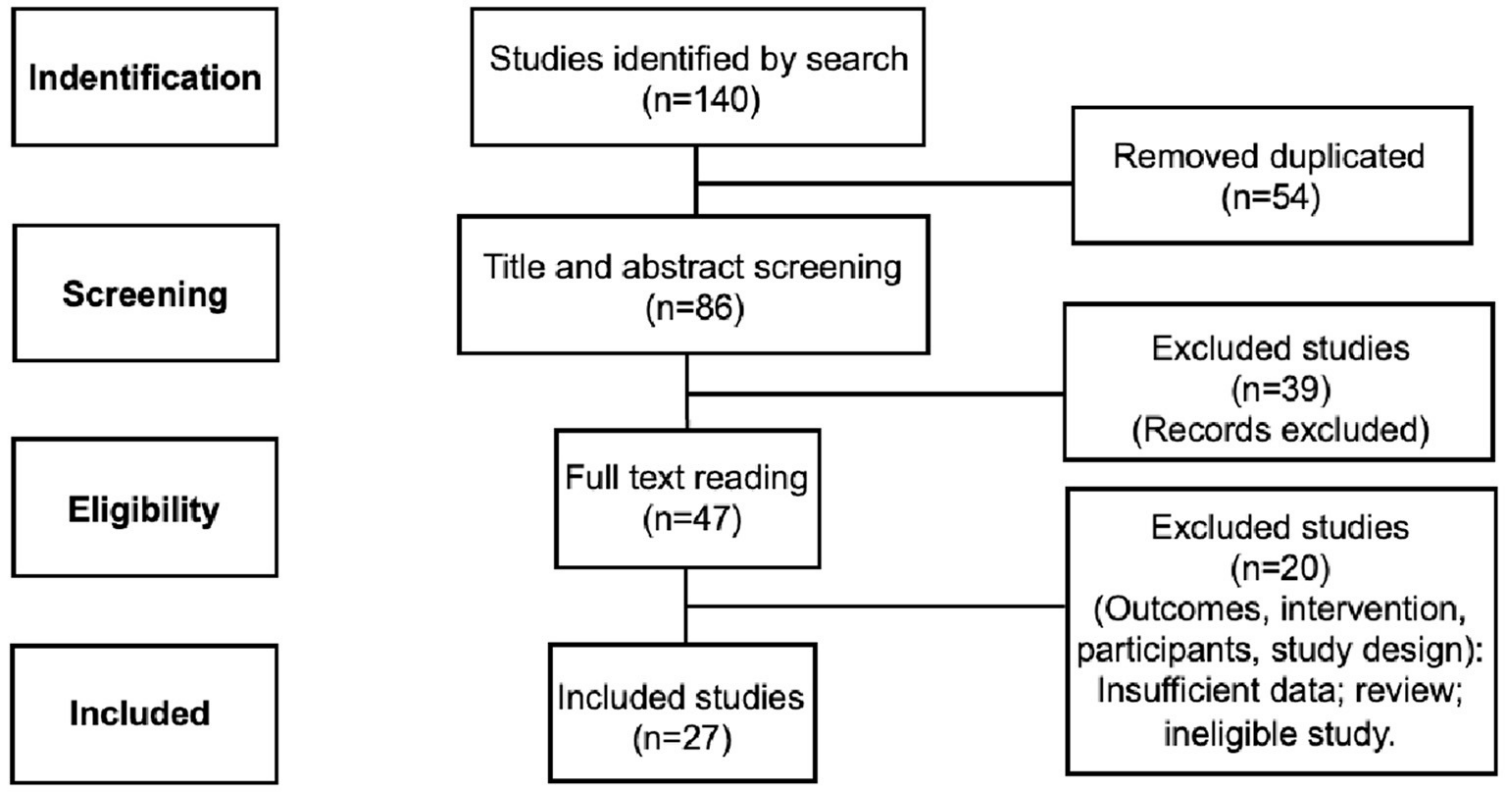
Diagram of article selection process.

The initial systematic search in PubMed and ScienceDirect databases identified 140 articles. Among them, 54 articles were removed for duplication and 59 articles by screening selection criteria. Then, 27 studies met the inclusion criteria (Figure 1).

### Characteristics of the studies

Twenty seven articles reported experiments in rodents, of which 3 were performed on albino BALB/c mice (Salehpour et al., 2019a,b; Hosseini et al., 2022), 6 used C57BL/6 mice (Khuman et al., 2012; Zhang et al., 2014; Gonçalves et al., 2016; Lee et al., 2016, 2017; Duarte et al., 2018), 1 was conducted on the 5XFAD transgenic mice (pigmented C57BL/6 background) (Cho et al., 2020), 1 was performed on the APP/PS1 transgenic mice (Wu et al., 2021), 2 used the TgF344 transgenic mice (Yang et al., 2021b, 2022), 6 were performed using Wistar rats (Moreira et al., 2009; Prianti et al., 2014; Cardoso et al., 2021a, 2022; Gerace et al., 2021; Vogel et al., 2021), and 8 used Sprague-Dawley rats (Lu et al., 2017; Esenaliev et al., 2018; Yang et al., 2018, 2021a; O'Brien and Austin, 2019; Di Paolo, 2021; Wang et al., 2021; Tsai et al., 2022). In these laboratory rodent studies, the age of animals varied from 7 weeks old to 20 months old. In order to analyze the anti-inflammatory effects of PBM on brain, we reviewed various animal models such as focal brain damage (Moreira et al., 2009), controlled cortical impact (Khuman et al., 2012), peripheral inflammation (Prianti et al., 2014), mild traumatic brain injury (Zhang et al., 2014), spinal cord injury (Wang et al., 2021), Aβ-treatment (Lu et al., 2017), Aβ and PS1 transgenic rodents (Cho et al., 2020), PS1 transgenic rodents (Wu et al., 2021), TgF344 transgenic rodents (Yang et al., 2021b, 2022), brain ischemia (Lee et al., 2016, 2017; Gerace et al., 2021; Vogel et al., 2021), multiple sclerosis (Gonçalves et al., 2016), natural aging (Cardoso et al., 2021a, 2022), blast injury (Esenaliev et al., 2018), retinal degeneration (Di Paolo, 2021), induced aging (Hosseini et al., 2022), cuprizone-induced demyelination (Duarte et al., 2018), photothrombotic stroke (Yang et al., 2018), lipopolysaccharide-induced Parkinson's disease (O'Brien and Austin, 2019), experimental model of epilepsy (Tsai et al., 2022), restraint stress-induced depression experimental model (Salehpour et al., 2019a), transient global brain ischemia and artificially aging (Salehpour et al., 2019b), and neonatal hypoxic ischemia (Yang et al., 2021a).

The laser parameters used in the studies showed wide divergence:

**Light source/ wavelength (nm):** LED and LASER, 610 to 905;

**Operation mode:** CW and pulsed;

**Output power (W):** 0.03 to 1.91;

**Irradiance (mW/cm**^**2**^**):** 0.457 to 100;

**Irradiation time per session (s):** 3 to 3,600;

**Total fluence (J/cm**^**2**^**):** 1.0 to 535.7;

**Energy (J):** 0.6 to 294;

**Irradiation approach/sites:** contact to the rat skin immediately over the lesion site, transcranial, in the open craniotomy, in the spinal cord, on the abdomen of pregnant rats, in the cells and percutaneous.

**Number of treatment sessions:** 1 to 207.

The selected studies were summarized in a chronological order shown in Table 2.

**Table 2:** Summary of laboratory animal studies on neuroinflammatory effects of the photobiomodulation.

**Author**	**Animal/Species**	**Model**	**Laser Parameters**	**Outcomes**
Moreira et al. (2009)	Fifty-one Male Wistar rats	Focal brain damage	**Light source/ wavelength (nm):** LED, 660 and 780 **Operation mode:** CW **Output power (W):** 0.04 **Irradiation time per session (s):** 3 and 5 **Energy (J):** 24 and 40 **Irradiation approach/sites:** contact to the rat skin immediately over the lesion site. **Number of treatment sessions:** 2	Modulation of TNF-α, IL-1β and IL-6 levels in the brain and in circulation in the first 24 h following cryogenic brain injury.
Khuman et al. (2012)	Two hundred and thirty-nine Male C57BL/6 mice (3 months old)	Controlled cortical impact	**Light source/ wavelength (nm):** LASER, 800 **Output power (W):** 0.33, 0.65, 1.3 **Irradiance (mW/cm**^**2**^**):** 250, 500 and 1,000 **Irradiation time per session (s):** 120 and 420 **Total fluence (J/cm**^**2**^**):** 30, 60, 105, 120 and 210 **Energy (J):** 39, 78, 137, 156 and 273 **Irradiation approach/sites**: in the open craniotomy and transcranial **Number of treatment sessions:** 1 and 7	Reduction of microgliosis in open craniotomy mice.
Prianti et al. (2014)	Thirty Male Wistar rats	Peripheral inflammation	**Light source/ wavelength (nm):** LED, 660 **Output power (W):** 0.03 **Irradiation time per session (s):** 232 **Total fluence (J/cm**^**2**^**):** 7.5 **Number of treatment sessions:** 1	Reduced expression of COX-2 mRNA.
Zhang et al. (2014)	Male C57BL/6 mice (8 weeks old)	Mild traumatic brain injury	**Light source/ wavelength (nm):** LED, 810 **Operation mode:** pulsed **Irradiance (mW/cm**^**2**^**):** 150 **Irradiation time per session (s):** 240 **Total fluence (J/cm**^**2**^**):** 36 **Irradiation approach/sites:** transcranial	Suppressed proinflammatory cytokine expression like IL-1b and IL-6.
Lee et al. (2016)	Eighteen Male C57BL/6J mice	Focal cerebral ischemia	**Light source/ wavelength (nm):** LED, 610 **Operation mode :** CW **Irradiance (mW/cm**^**2**^**) :** 1.7 **Total fluence (J/cm**^**2**^**):** 2.0 **Irradiation approach/sites:** transcranial **Number of treatment sessions:** 4	Inhibited Iba-1- and GFAP-labeled cells, which was accompanied by a reduction in the expression of inflammatory mediators and inhibition of MAPK activation and NF-kB translocation in the ischemic cortex.
Gonçalves et al. (2016)	Female C57BL/6 mice (6–10 weeks old)	Multiple sclerosis	**Light source/ wavelength (nm):** LED/ 660 and 904 **Operation mode:** CW and Pulsed **Output power (W):** 0.3 and 0.7 **Irradiation time per session (s):** 120 **Total fluence (J/cm**^**2**^**):** 3 and 10 **Energy (J):** 0.6 **Irradiation approach/sites:** in the spinal cord **Number of treatment sessions:** 30	Neuroinflammation inhibition/modulation through a reduction of inflammatory cells in the CNS.
Lee et al. (2017)	Male C57BL/6J mice	Focal cerebral ischemia	**Light source/ wavelength (nm):** LED, 610 **Irradiance (mW/cm**^**2**^**):** 1.7 **Irradiation time per session (s):** 1,200 **Total fluence (J/cm**^**2**^**):** 2 **Irradiation approach/sites:** transcranial **Number of treatment sessions:** 6	Attenuation of the NLRP3 inflammasome, in accordance with down- regulation of pro-inflammatory cytokines IL-1β and IL-18 in the ischemic brain. In addition, suppressed TLR-2 levels, MAPK signaling and NF-kB activation in the mice with post-is- chemic.
Lu et al. (2017)	Male Sprague- Dawley rats	Aβ-treatment	**Light source/ wavelength (nm):** LED, 808 **Operation mode :** CW **Irradiance (mW/cm**^**2**^**) :** 25 **Irradiation time per session (s):** 120 **Total fluence (J/cm**^**2**^**):** 15 **Irradiation approach/sites:** transcranial **Number of treatment sessions:** 5	Attenuation of the elevation of glial activation and proinflammatory cytokine levels (IL-1β, IL-6 and TNF-α,) in the hippocampal CA1 region.
Esenaliev et al. (2018)	Fifty Male Sprague-Dawley rats	Blast injury	**Light source/ wavelength (nm):** LASER, 808 **Operation mode:** Pulsed **Irradiation time per session (s):** 300 **Total fluence (J/cm**^**2**^**):** 300 **Irradiation approach/sites:** transcranial **Number of treatment sessions:** 1	Inhibition of microglia activation and reduction of the number of cortical neurons expressing activated caspase-3.
Duarte et al. (2018)	Male C57BL/6 mice (7 weeks old)	Demyelination	**Light source/ wavelength (nm):** LED, 808 **Operation mode:** CW **Output power (W):** 0.5 **Irradiance (mW/cm**^**2**^**):** 178 **Irradiation time per session (s):** 20 **Total fluence (J/cm**^**2**^**):** 36 **Energy (J):** 1 **Irradiation approach/sites:** transcranial **Number of treatment sessions:** 6	Modulation in microglial and astrocytes activation induced by cuprizone.
Yang et al. (2018)	Male Sprague-Dawley rats	Photothrombotic stroke	**Light source/ wavelength (nm):** LASER, 808 **Operation mode :** CW **Irradiance (mW/cm**^**2**^**) :** 350 **Irradiation time per session (s):** 120 **Energy (J):** 294 **Irradiation approach/sites:** transcranial **Number of treatment sessions:** 7	Modulation of M1 microglial phenotype to an anti-inflammatory M2 phenotype.
O'Brien and Austin (2019)	Forty-one Male Sprague–Dawley rats	Local inflammation and microglial activation	**Light source/ wavelength (nm):** LED, 675 **Operation mode:** CW **Output power (W):** 0.5 **Irradiance (mW/cm**^**2**^**):** 40.84 **Irradiation time per session (s):** 88 **Total fluence (J/cm**^**2**^**):** 3.594 **Energy (J):** 35.94 **Irradiation approach/sites:** transcranial **Number of treatment sessions:** 13	Protection against a dose of LPS sufficient to cause 15% dopaminergic cell death.
Salehpour et al. (2019a)	Seventy-five Male BALB/c mice (8–10-weeks-old)	Restraint stress	**Light source/ wavelength (nm):** LASER, 810 **Operation mode:** Pulsed **Output power (W):** 0.2 **Irradiance (mW/cm**^**2**^**):** 666 **Irradiation time per session (s):** 5 **Total fluence (J/cm**^**2**^**):** 33.3 **Energy (J):** 1 **Irradiation approach/sites:** transcranial **Number of treatment sessions:** 5	Suppression of neuroinflammatory response in the cortex and hippocampus by decreased NF-kB, p38, and JNK levels. In addition, decreased the serum levels of cortisol, corticosterone, TNF-α, and IL-6 induced by restraint stress.
Salehpour et al. (2019b)	Ninety Male BALB/c mice (8–10- weeks old)	Transient global brain ischemia in artificially aged	**Light source/ wavelength (nm):** LASER, 810 **Operation mode:** Pulsed **Output power (W):** 0.2 **Irradiance (mW/cm**^**2**^**):** 666 **Total fluence (J/cm**^**2**^**):** 33.3 **Energy (J):** 1 **Irradiation approach/sites:** transcranial **Number of treatment sessions:** 14	Reduction of iNOS, TNF-α, and IL-1β brain levels.
Cho et al. (2020)	5XFAD transgenic male mice (10 months old)	Aβ and OS1 treated	**Light source/ wavelength (nm):** LED, 610 **Irradiance (mW/cm**^**2**^**):** 1.7 **Irradiation time per session (s):** 1,200 **Total fluence (J/cm**^**2**^**):** 2.0 **Irradiation approach/sites:** transcranial **Number of treatment sessions:** 42	Reduced microglia (Iba-1 immunoreactivity) in the cerebral cortex.
Cardoso et al. (2021a)	Sixty-four Male Wistar rats (4 and 20 months old)	Aging	**Light source/ wavelength (nm):** LED, 810 **Operation mode:** CW **Output power (W):** 0.1 **Irradiance (mW/cm**^**2**^**):** 357 **Irradiation time per session (s):** 150 **Total fluence (J/cm**^**2**^**):** 535.7 **Energy (J):** 15 **Irradiation approach/sites:** transcranial **Number of treatment sessions:** 58	Increased cerebral cortex levels of IL-10, IL-6, and TNFα, and decreased IL-5. Also, decreased hippocampal levels of IP-10 and fractalkine.
Yang et al. (2021a)	Thirty Male and female Sprague- Dawley rats (11 weeks old)	Neonatal hypoxic ischemia	**Light source/ wavelength (nm):** LED, 808 **Irradiance (mW/cm**^**2**^**):** 350 and 8 on neonatal brain **Irradiation time per session (s):** 120 **Irradiation approach/sites:** on the abdomen of pregnant rats **Number of treatment sessions:** 9	Settled hypoxic-ischemic-induced neuroinflammation, oxidative stress, and myeloid cell/astrocyte activation.
Wu et al. (2021)	Ninety-six APP/PS1 transgenic mice (6 months old)	OS1 treatment	**Light source/ wavelength (nm):** LED, 635 **Output power (W):** 0.1 **Irradiance (mW/cm**^**2**^**):** 12.74 **Irradiation time per session (s):** 75, 150 and 300 **Total fluence (J/cm**^**2**^**):** 1, 2 and 4 **Irradiation approach/sites:** in the cells **Number of treatment sessions:** 1	Expression of glial fibrillary acidic protein (GFAP) inhibition.
Vogel et al. (2021)	Fifty Male Wistar rats	Ischemic stroke	**Light source/ wavelength (nm):** LED, 780 **Output power (W):** 0.150 **Irradiance (mW/cm**^**2**^**):** 10 **Irradiation time per session (s):** 120 **Total fluence (J/cm**^**2**^**):** 10 **Irradiation approach/sites:** transcranial **Number of treatment sessions:** 25	Reduced of TNF-α, IL-1β and IL-6 and microglial activation.
Wang et al. (2021)	Two hundred and seventy-nine Male Sprague- Dawley rats	Spinal cord injury	**Light source/ wavelength (nm):** LED, 810 **Operation mode:** CW **Output power (W):** 1.0 **Irradiation time per session (s):** 3600 **Irradiation approach/sites:** percutaneous **Number of treatment sessions:** 14	Inhibition of the activation of neurotoxic microglia, neuroinflammation alleviation.
Yang et al. (2021b)	Thirty-two Male TgF344 rats (2 months old)	A4 and PS1 treatment	**Light source/ wavelength (nm):** LED, 808 **Operation mode:** CW **Irradiance (mW/cm**^**2**^**):** 350 **Irradiation time per session (s):** 120 **Irradiation approach/sites:** transcranial **Number of treatment sessions:** 103	Neuroinflammation and oxidative stress decrease.
Di Paolo (2021)	Male Sprague- Dawley rats	Retinal Degeneration	**Light source/ wavelength (nm):** LED, 670 **Irradiation time per session (s):** 180 **Total fluence (J/cm**^**2**^**):** 4.5 **Irradiation approach/sites:** transcranial **Number of treatment sessions:** 7	Mitigation of the microglial activation.
Gerace et al. (2021)	Male and female Wistar rats (7–9 days old)	Cerebral Hypoxia/Ischemia	**Light source/ wavelength (nm):** LED, 808 and 905 **Operation mode:** CW and pulsed **Output power (W):** 1.91 **Irradiance (mW/cm**^**2**^**):** 620 **Irradiation time per session (s):** 6, 12 e 24 **Total fluence (J/cm**^**2**^**):** 3.71, 7.42 and 14.84 **Irradiation approach/sites:** in the cells **Number of treatment sessions:** 1	Attenuation of inflammatory mechanisms.
Hosseini et al. (2022)	Fifty Male BALB/c mice	Aging	**Light source/ wavelength (nm):** LED, 810 **Output power (W):** 0.2 **Irradiance (mW/cm**^**2**^**):** 0.457 **Irradiation time per session (s):** 5, 10 and 20 **Total fluence (J/cm**^**2**^**):** 8, 16 and 32 **Irradiation approach/sites:** transcranial **Number of treatment sessions:** 24	Decrease of TNF-α and IL-6; down-regulation of GAP-43 and SYN inhibition.
Cardoso et al. (2022)	Ten Male Wistar rats (20 months old)	Aging	**Light source/ wavelength (nm):** LED, 660 **Operation mode:** CW **Output power (W):** 0.1 **Irradiance (mW/cm**^**2**^**):** 357 **Irradiation time per session (s):** 150 **Total fluence (J/cm**^**2**^**):** 535.7 **Energy (J):** 15 **Irradiation approach/sites:** transcranial **Number of treatment sessions:** 10	Increased levels of IL-1α and decreased levels of IL-5 in the cerebral cortex. In the hippocampus, the laser treatment increased the levels of IL-1α and decreased levels of IL-5, IL-18, and fractalkine.
Tsai et al. (2022)	Male Sprague- Dawley rats	Epilepsy	**Light source/ wavelength (nm):** LED, 808 **Operation mode:** CW **Output power (W):** 0.11 **Irradiance (mW/cm**^**2**^**):** 133.3 **Irradiation time per session (s):** 100 **Total fluence (J/cm**^**2**^**):** 133.3 **Energy (J):** 11 **Irradiation approach/sites:** transcranial **Number of treatment sessions:** 1	Reduced NSE immunoreactivity in CA3, GFAP immunoreactivity in CA1, and Iba-1 immunoreactivity in CA3.
Yang et al. (2022)	Sixty-four Male TgF344 rats (2 months old)	A4 and PS1 treatment	**Light source/ wavelength (nm):** LED, 808 **Operation mode:** CW **Irradiance (mW/cm**^**2**^**):** 350 **Irradiation time per session (s):** 120 **Total fluence (J/cm**^**2**^**):** 42 **Irradiation approach/sites:** transcranial **Number of treatment sessions:** 207	Regulation of glial cell polarization and inhibition of neuroinflammation.

## Findings and discussion

The purpose of this systematic review was to investigate the neuroinflammatory effects of PBM therapy. Studies have shown interesting findings on the anti-inflammatory effects of PBM in various animal models of neurological diseases in different neurological conditions, such as traumatic brain injury, edema formation and hyperalgesia, ischemia, neurodegenerative conditions, aging, and depression.

### Traumatic brain injury

Studies using laboratory animals have shown that PBM reduces the level of pro-inflammatory cytokines and the microglia activation of TBI animal models (Moreira et al., 2009; Khuman et al., 2012; Esenaliev et al., 2018; Yang et al., 2018). For example, Khuman et al. (2012) demonstrated that 800 nm laser PBM inhibited the microglia activation, accompanied by improvement in cognitive deficits after controlled cortical impact. Esenaliev et al. (2018) observed that one session of 808 nm nano-pulsed laser PBM therapy applied 1 h after blast injury significantly inhibited microglia activation and reduced the number of cortical neurons expressing activated caspase-3 in a rat model of blast-induced neurotrauma. Also, in the study conducted by Yang et al. (2018), 9 sessions of 808 nm laser PBM was able to change the phenotype of microglial polarization from the M1 pro-inflammatory phenotype to the M2 anti-inflammatory phenotype. Since TBI is accompanied by an increase in cytokine and chemokine levels (Woodcock and Morganti-Kossmann, 2013; Bergold, 2016), the severity of brain damage is linked to a higher and more prolonged inflammatory response (Kumar and Loane, 2012; White et al., 2013; Woodcock and Morganti-Kossmann, 2013; Lozano et al., 2015). In animals, an increase in cerebral cortical levels of the inflammatory cytokines IL-1β, TNFα, and IL-6 has been shown from 3 to 9 h after injury (Bachstetter et al., 2013). In clinical studies, levels of pro-inflammatory markers IL-6, TNFα, IL-10, IL-8, and monocyte chemoattractant protein-1 (MCP-1) have also been increased after 2 days of TBI (Morganti-Kossman et al., 1997; Csuka et al., 1999; Semple et al., 2010). In addition, the release of these cytokines was correlated with microglial activation and axonal dysfunction, suggesting an association between the activated immune response and brain injury (Frugier et al., 2010). Taken together, it seems that PBM can exert anti-inflammatory action against TBI through the modulation of both anti- and pro-inflammatory chemokines and cytokines.

### Edema formation and hyperalgesia

In the study by Prianti et al. (2014), they showed that 660 nm laser PBM reduces COX-2 mRNA expression in animals receiving carrageenan. This is a worthwhile result as in inflammatory conditions COX-2 is highly expressed, increasing the release of pro-inflammatory markers (Schuligoi et al., 2003; Grill et al., 2006, 2008), and plays a key role in chronic pain (Narita et al., 2008).

### Brain ischemia

Ischemia can trigger an imbalance between pro- and anti-inflammatory mediators (Yilmaz and Granger, 2008), which play a key role in the progression and pathogenesis of ischemia (Barone and Feuerstein, 1999; Samson et al., 2005; Chamorro and Hallenbeck, 2006; Wang et al., 2007). For example, the inhibition of inflammatory response in ischemic patients can decrease the brain injury (Yilmaz and Granger, 2008). The therapeutic effects of PBM on ischemia have been addressed in the works by Zhang et al. (2014), Lee et al. (2016, 2017), Salehpour et al. (2019b), Vogel et al. (2021), Yang et al. (2021a) and Gerace et al. (2021). Lee et al. (2016, 2017) treated mice submitted to a focal brain ischemia experimental model with a 610 nm laser and observed an inhibition of Iba-1 and GFAP-labeled cells, accompanied by a regulation of pro-inflammatory cytokines and suppression of mitogen activated protein kinase (MAPK) (a signaling pathway linked to inflammation and cell death) and NF-kB activation. Salehpour et al. (2019b) showed that 14 sessions of 810 nm laser PBM significantly decreased iNOS, TNF-α, and IL-1β levels in the brain of transient global cerebral cortex ischemia model using artificially aged mice. This evidence is promising since activation of MAPKs p38, ERK and JNK regulate pro-inflammatory genes that activate NF-kB in microglia.

### Neurodegenerative conditions

Abnormal microglial activation and inflammatory response may contribute to the pathology of several neurodegenerative conditions (Chen et al., 2016; Swaroop et al., 2016; Shabab et al., 2017; Voet et al., 2019). It is known that patients with multiple sclerosis (Huang et al., 2020), Alzheimer's disease (Licastro et al., 2000), and Parkinson's disease (Liu et al., 2003) exhibit elevated levels of pro-inflammatory markers in plasma. Transcranial PBM therapy has anti-inflammatory effects in several models of neurodegenerative conditions (Gonçalves et al., 2016; Lu et al., 2017; Duarte et al., 2018; O'Brien and Austin, 2019; Cho et al., 2020; Di Paolo, 2021; Wu et al., 2021; Yang et al., 2021b, 2022). For instance, Gonçalves et al. (2016) submitted mice to a model of multiple sclerosis to 30 sessions of either 660 nm or 904 nm laser PBM therapy. PBM-treated mice exhibited decreased levels of IL-1β, IL-17 and interferon-γ (IFN-γ) in the spinal cord, when compared to the control group mice. In addition, Duarte et al. (2018) demonstrated that 6 sessions of 808 nm laser PBM modulate microglial and astrocytes activation induced by cuprizone. In Aβ-treated mice, PBM treatment also reduced microglia (Iba-1 immunoreactivity) in the cerebral cortex (Cho et al., 2020), and attenuated the elevation of glial activation and IL-1β, IL-6, and TNF-α levels in the hippocampal CA1 region (Lu et al., 2017). Furthermore, O'Brien and Austin (2019) observed that PBM protected against lipopolysaccharide-induced dopaminergic cell death in a rat Parkinson's disease experimental model.

### Aging

Brain aging is characterized by microglia reactivity and an imbalance between pro- and anti-inflammatory cytokines (Godbout and Johnson, 2009; Jurgens and Johnson, 2012). However, a couple of recent studies involving our group have shown that PBM can improve the inflammatory response in the aging brain (Salehpour et al., 2019b; Cardoso et al., 2021a, 2022; Hosseini et al., 2022). For example, a protocol with 58 consecutive days of 810 nm laser PBM therapy was able to change the inflammatory profile of the aging brain in rats. We showed that PBM increased cerebral cortex levels of IL-10, IL-6, and TNF-α. In addition, PBM therapy significantly decreased cerebral cortex levels of IL-5 and hippocampal levels of IP-10 and fractalkine (Cardoso et al., 2021a). In addition, we reported that 10 sessions of 660 nm laser PBM Increased levels of IL-1α and decreased levels of IL-5 in the cerebral cortex. In the hippocampus, the laser treatment increased the levels of IL-1α and decreased levels of IL-5, IL-18, and fractalkine (Cardoso et al., 2022). These findings are promising, since the expression of pro-inflammatory cytokines, oxidative stress, and glial activation are increased during the aging (Lee et al., 2000; Blalock et al., 2003; Godbout et al., 2005; Bishop et al., 2010).

### Epilepsy

In the study conducted by Tsai et al. (2022), they showed that 608 nm laser PBM reduced neuron-specific enolase (NSE) and glial fibrillary acid protein (GFAP) immunoreactivity in hippocampus in an animal model of epilepsy. These results are promising since studies suggest inflammation as a biomarker in epilepsy (Ravizza et al., 2008; Auvin et al., 2010; Vezzani and Friedman, 2011; Vezzani et al., 2016). For example, blocking IL-1β (a pro-inflammatory interleukin) prevents generalized seizure and increases the threshold for induction of afterdischarge (Ravizza et al., 2008; Auvin et al., 2010).

### Depression

Salehpour et al. (2019a) observed that 5 sessions of 810 nm laser PBM suppressed neuroinflammatory responses in the neocortex and hippocampus of mice submitted to a restraint stress-induced depression model by decreasing NF-kB, p38, and JNK levels. In addition, PBM decreased the serum levels of cortisol, corticosterone, TNF-α, and IL-6 induced by restraint stress. These results are promising since evidence suggests that these pro-inflammatory proteins are involved in the pathology of major depressive disorder (Kubera et al., 2011; Liu et al., 2012), as well as neurotransmission and mood regulation (Du et al., 2008; Villanueva, 2013).

### Spinal cord injury

In the study conducted by Wang et al. (2021) it was observed that microglia and astrocytes begin to be activated after spinal cord injury, participating in secondary damage and tissue repair. However, 810 nm laser PBM during two consecutive weeks was able to inhibit microglia/macrophage and astrocyte activation after spinal cord injury. In this regard, PBM may be a useful tool for the treatment of spinal cord injury, in association to antibody-based approaches to interrupt endothelial-monocyte interactions, reducing macrophage activation at the injured spinal cord and also to pharmacological therapies focusing on immunomodulation and promotion of reparative glia activity (Orr and Gensel, 2018).

### Limitations

Our review presents limitations. The researches highlighted in this review describe several brain conditions and methodologies, and the lack of some details about the PBM parameters used in each work make it difficult to replicate these approaches. Standardization of the protocols for each condition would facilitate comparison between the findings of the studies and could improve the translational application of PBM therapy.

## Conclusion

Neuroinflammation is a pathological condition in a variety of brain insults and neurodegenerative conditions. Despite using very different protocols, the reviewed studies showed that the therapeutic effects of transcranial PBM therapy in animal models of neurological and psychiatric diseases are related to the capacity to reduce levels of pro-inflammatory mediators and increase levels of anti-inflammatory mediators. In addition, no adverse effects of PBM on the brain were found. Therefore, PBM could safely fit in to complement current treatments for the conditions listed above. These results mean that for the current use of PBM, controlled human studies are needed as a next-step of research to build on these animal studies. Despite not excluding human studies as a keyword, no controlled human studies were discovered in the present review, and all the included studies were animal studies. Human studies could bring new perspectives on the anti-inflammatory property of PBM in brain disorders. The reviewed animal studies together with consistent human studies of PBM in the treatment of neuroinflammation can suggest that transcranial PBM is a promising strategy for the treatment of neuroinflammation-induced brain diseases.

## Data availability statement

The raw data supporting the conclusions of this article will be made available by the authors, without undue reservation.

## Author contributions

All authors listed have made a substantial, direct, and intellectual contribution to the work and approved it for publication.

## Funding

This study was supported by Coordenação de Aperfeiçoamento de Pessoal de Nível Superior (CAPES) and Fundação de Amparo à Pesquisa do Estado de São Paulo (FAPESP) (grants 2017/16443-0, 2021/06473-4, and 2020/15050-7). FG-L was supported by the Oskar Fischer Project Fund.

## Conflict of interest

The authors declare that the research was conducted in the absence of any commercial or financial relationships that could be construed as a potential conflict of interest.

## Publisher's note

All claims expressed in this article are solely those of the authors and do not necessarily represent those of their affiliated organizations, or those of the publisher, the editors and the reviewers. Any product that may be evaluated in this article, or claim that may be made by its manufacturer, is not guaranteed or endorsed by the publisher.
