# Supplementary figures and images for: Photobiomodulation for the treatment of neuroinflammation: A systematic review of controlled laboratory animal studies
Source: Front Neurosci. 2022 Sep 20;16:1006031. doi: 10.3389/fnins.2022.1006031 (PMC9531128; doi:10.3389/fnins.2022.1006031)

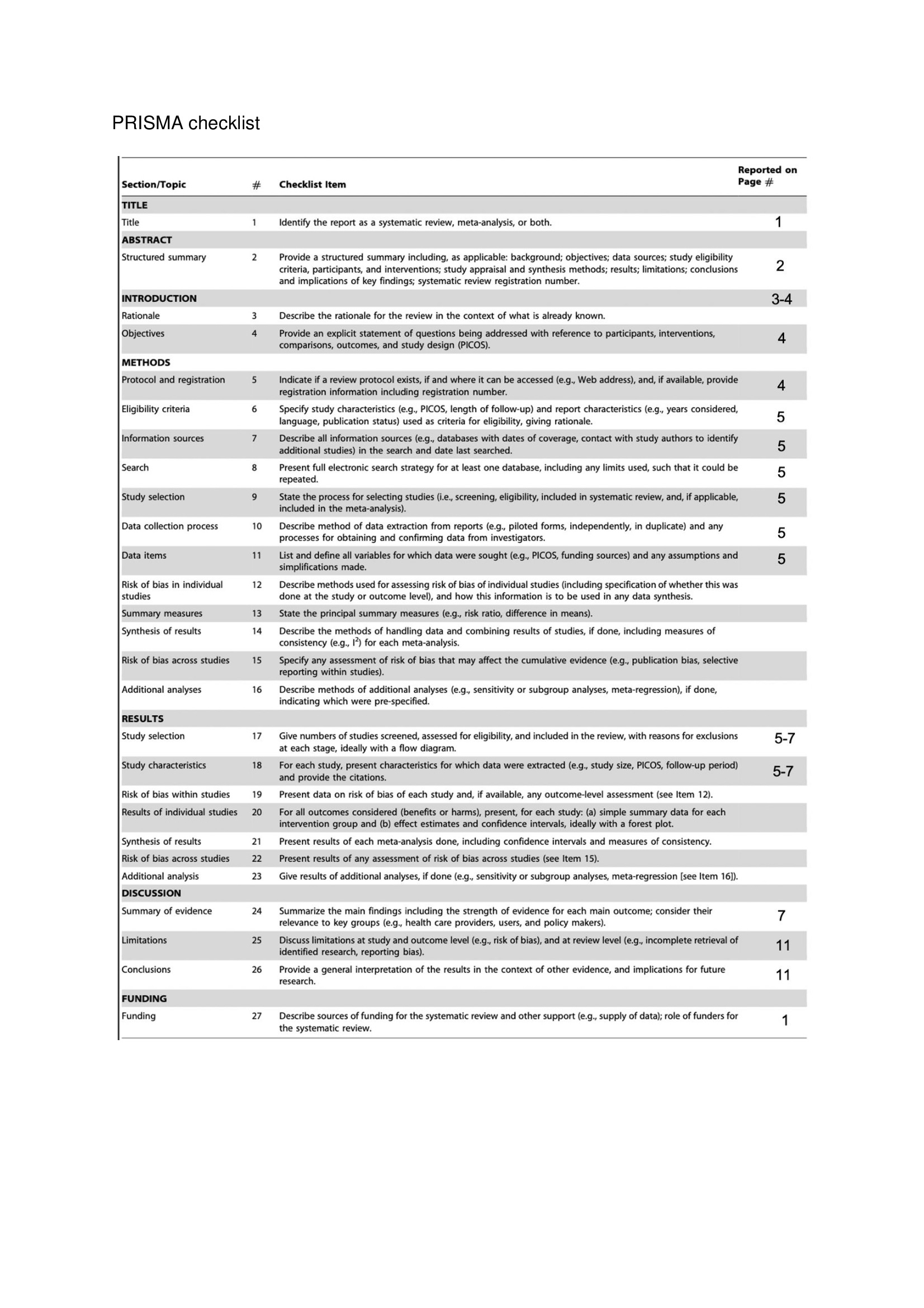

Supplement: Supplementary file 1 [file Image_1.JPG]
